# Knowledge about tuberculosis transmission and prevention and perceptions of health service utilization among index cases and contacts in Brazil: Understanding losses in the latent tuberculosis cascade of care

**DOI:** 10.1371/journal.pone.0184061

**Published:** 2017-09-21

**Authors:** Flavia Matos Salame, Márcia Danielle Ferreira, Marcia Teresa Belo, Eleny Guimarães Teixeira, Marcelo Cordeiro-Santos, Ricardo Arraes Ximenes, Maria de Fátima Militão de Albuquerque, Philip C. Hill, Dick Menzies, Anete Trajman

**Affiliations:** 1 Fundação de Medicina Tropical Dr. Heitor Vieira Dourado, Manaus, AM, Brazil; 2 Universidade do Estado do Amazonas, Manaus, AM, Brazil; 3 Fundação Técnico-Educacional Souza Marques, Rio de Janeiro, RJ, Brazil; 4 Universidade Federal de Pernambuco, Recife, PE, Brazil; 5 Instituto de Pesquisa Aggeu Magalhães, Fiocruz, Recife, PE, Brazil; 6 Centre for International Health, University of Otago, Dunedin, New Zealand; 7 Global Health Program, McGill University, Montreal, Canada; 8 Instituto de Medicina Social, Universidade do Estado do Rio de Janeiro, RJ, Brazil; Indian Institute of Technology Delhi, INDIA

## Abstract

**Introduction:**

Tuberculosis contacts are candidates for active and latent tuberculosis infection screening and eventual treatment. However, many losses occur in the different steps of the contacts’ cascade of care. Reasons for this are poorly understood.

**Objective:**

To describe the different steps where losses in the contact cascade occur and to explore knowledge and attitudes regarding tuberculosis transmission/prevention and perceptions about tuberculosis services in order to understand the reasons for losses from the tuberculosis service users’ perspective.

**Design:**

We collected routine data from the index case and contact registry books and from patients’ records to build the cascade of care of contacts in 12 health facilities in three Brazilian cities with high tuberculosis incidence rates. During a knowledge, attitudes and practices (KAP) survey, trained interviewers administered a semi-structured questionnaire to 138 index cases and 98 contacts.

**Results:**

Most of the losses in the cascade occurred in the first two steps (contact identification, 43% and tuberculin skin testing placement, 91% of the identified contacts). Among KAP-interviewed contacts, 67% knew how tuberculosis is transmitted, 87% knew its key symptoms and 81% declared they would take preventive therapy if prescribed. Among KAP-interviewed index cases, 67% knew they could spread tuberculosis, 70% feared for the health of their families and 88% would like their family to be evaluated in the same services.

**Conclusion:**

Only a small proportion of contacts are evaluated for active and latent tuberculosis, despite their—and their index cases’—reasonable knowledge, positive attitudes towards prevention and satisfaction with tuberculosis services. In these services, education of service users would not be a sufficient solution. Healthcare workers’ and managers’ perspective, not explored in this study, may bring more light to this subject.

## Introduction

The World Health Organization (WHO) considers investigation of tuberculosis (TB) index case contacts a fundamental activity to control TB,[1] a disease that still affects approximately 10.4 million people and kills 1.4 million a year globally.[2] Contact investigation helps to identify active TB disease and latent *Mycobacterium tuberculosis* infection (LTBI) in contacts.[3] Those with LTBI are at high risk for progression to active disease, thus they are an important reservoir for new TB cases.[4] The treatment of LTBI with isoniazid (INH) substantially reduces this risk by up to 90%.[5]

Although prophylactic regimens have been recommended by the American Thoracic Society and by WHO since 1970's,[6] under routine conditions rates of LTBI treatment completion are low.[7] Non-adherence to treatment is just a part of the problem. A recent systematic review on the cascade of care in diagnosis and treatment of LTBI confirmed that important losses occur at each of the steps of initial screening, completing medical evaluation, and starting therapy.[8] This phenomenon was observed in both high-income[9] and low- and medium-income countries.[10] Thus, actions to prevent losses in the pre-treatment steps could likely result in more impact in LTBI treatment outcomes than non-adherence problems.

Few studies have explored the extent, risk factors and reasons for the losses in the pre-treatment LTBI phase from the patients’ and contacts’ perspective.[11,12] Most knowledge, attitudes and practice (KAP) surveys on LTBI have focused on health professionals,[13,14] on high-risk populations (such as, the immigrants,[15] the homeless[16] and the African-Americans[17]) and on difficulties in adherence to treatment and side effects of medications.[18] To our knowledge, no studies have explored this issue from the contacts’ perspective and only two from the perspective of health services users (herein called users).[17,19]

In Brazil, the Ministry of Health currently recommends treating LTBI in all eligible TB contacts, i.e., contacts of any age with a positive tuberculin skin test (TST) and no signs of active TB on chest radiograph, with INH for six to nine months.[20] Thus, contact investigation in Brazil should start with an interview to detect symptoms and a TST. Those with symptoms or a positive TST should undergo full medical evaluation, including a chest radiograph. A recent study in Rio de Janeiro found that, among 1078 pediatric contacts, the most important losses occurred before INH prescription (206/322 with tuberculin skin test -TST- positive, 64%), in the investigation process, while 63% of those prescribed INH (73/115) completed treatment.[10] Healthcare workers attribute low attendance to LTBI programs in Brazil to insufficient knowledge, unaffordable costs and lack of interest in contacts,[21–23] but this remains to be proven. Therefore, we evaluated the cascade of LTBI care among Brazilian contacts and conducted a KAP survey regarding TB transmission and prevention, perceptions about health services utilization and patients’ health costs in three state capitals with high TB incidence rates: Recife (78.3/100,000 population/year), Rio de Janeiro (66.8/100,000) and Manaus (98.3/100,000).[24]

## Methods

### Study design

This was an observational cross-sectional study. For the survey, we used a Knowledge, Attitudes and Practice (KAP) design, a representative study of a specific population to collect information on what is known, believed (attitudes) and done (practice) in relation to a particular topic.[25,26]^,^ In most KAP studies, data are collected by an interviewer using a structured, standardized questionnaire. These data can then be analyzed in a quantitative or qualitative format, according to research objectives. A KAP survey may be specially designed to collect information about a topic, but it is also possible to include general questions about practices and beliefs.[26] No interventions were made by the research team as part of this study.

### Setting

The study was conducted in Brazil. Three cities among the four highest TB[27] incidence rates in the country were included: Manaus, Rio de Janeiro and Recife.

Brazil is an upper middle-income country with very sharp inequities in income and health. Its GINI index is 51.5.[28] It is among the top 30 high TB burden countries according to WHO.[2] TB treatment is restricted to public free-of-charge health clinics. Since 2010, the National TB Program recommends screening of LTBI for all contacts, regardless of age or co-morbidity. Contacts are screened for symptoms and a TST is performed in the initial visit. For those who are positive for any of the two, full medical evaluation including a chest radiograph are indicated. For those who are asymptomatic, TST-positive and no signs of active TB in the chest radiograph, LTBI treatment is recommended.[20]

### Identification of clusters

In the three participating capitals, we identified the number of new TB cases notified per health unit per year and selected, in each city, the four units with the highest number of cases to be included as data collection sites. Those were mostly primary care clinics with some having reference services for TB treatment.

### Data collection

Data regarding the steps of the cascade of care were collected from routine clinic sources. The national TB registry book (used by all clinics in the country), the TST book (used by some clinics) or index case and, when available, contact medical records and prescription lists and other sources of information routinely used by the clinics were consulted. Data were collected for the first semester of 2014, when there was full availability of PPD in the country (because of the shortage in the international market, PPD distribution was limited from July 1^st^ 2014).

A semi-structured questionnaire previously applied in Indonésia[29] addressing questions about TB transmission and prevention, attitudes and beliefs regarding LTBI and their perception regarding TB services was forward- and back-translated by independent translators and adapted to the Brazilian context.[30] Questions about payment of visits, diagnostic tests and TB medication, for example, were suppressed, since TB care is only provided in public free-of-charge clinics, where we conducted the interviews. Open-ended questions were mostly objective, no options of answers were offered. The questionnaire was tested and refined in a pilot study done in Rio de Janeiro approaching 28 index cases and 19 contacts. During this pilot, we labeled and grouped answers, until new responses appeared very infrequently. For example, LTBI is diagnosed by TST, by PPD, by a test in the skin, were all considered as “diagnosed by TST”. During this whole process, responses not previously included were added.

The entire interview process took place from March 11, 2015 to October 21, 2015, during different periods in each city (because of local ethical clearance). Interviewers were previously trained to standardize the approach to the participants for consent and the application of the questionnaire. The training was carried out approximately one month before the beginning of the data collection by the principal investigator of the research project in Brazil (AT), in each of the three participating cities. The teams comprised two to three interviewers in each city. During the training, interviews supervised by the principal investigator were conducted with volunteers.

### Participants

Patients treated for bacteriologically confirmed (smear microscopy, rapid molecular test or culture) pulmonary TB and their close contacts (those who shared an enclosed space, such as a social gathering place, workplace, or facility, with the index case for extended daytime periods during the 3 months before the start of the current treatment episode)[31] at the 12 health centers during the data collection period were eligible for the interview. Consenting close contacts were recruited regardless of their LTBI status. Those under 18 years were excluded. In the clinics, participants were interviewed in a private space (in general, a consultation room). The sample size at each clinic varied according to the number of staff, patients and contacts eligible during the time period of the study.

### Data analysis

Data were stored in a Microsoft Office Excel 2010 system spreadsheets. The frequencies, means and median values were calculated using the IBM SPSS Statistics software 23.

Answers were classified as satisfactory when "compulsory" responses were cited and if "unacceptable" answers were not mentioned. For example, knowledge about TB transmission was considered satisfactory if the respondent correctly identified the primary transmission route as airborne (i.e. “contact with someone with TB who sneezes, speaks or cough" or "living with someone who has TB") AND if they did not express misleading conceptions that could interfere with their practices (such as "It was a God’s decision"), even if (s)he also expressed other misconceptions (such as “using the same utensils”).

### Ethical approval

This study was approved in Rio de Janeiro on August 8^th^, 2014 (CAAE 762.361); in Manaus on March 25th, 2015 (CAAE 998.112) and in Recife on June 6^th^, 2015 (CAAE 1.097.557). All interviewees gave written informed consent.

## Results

### Cascade of LTBI care

**Fig 1:**
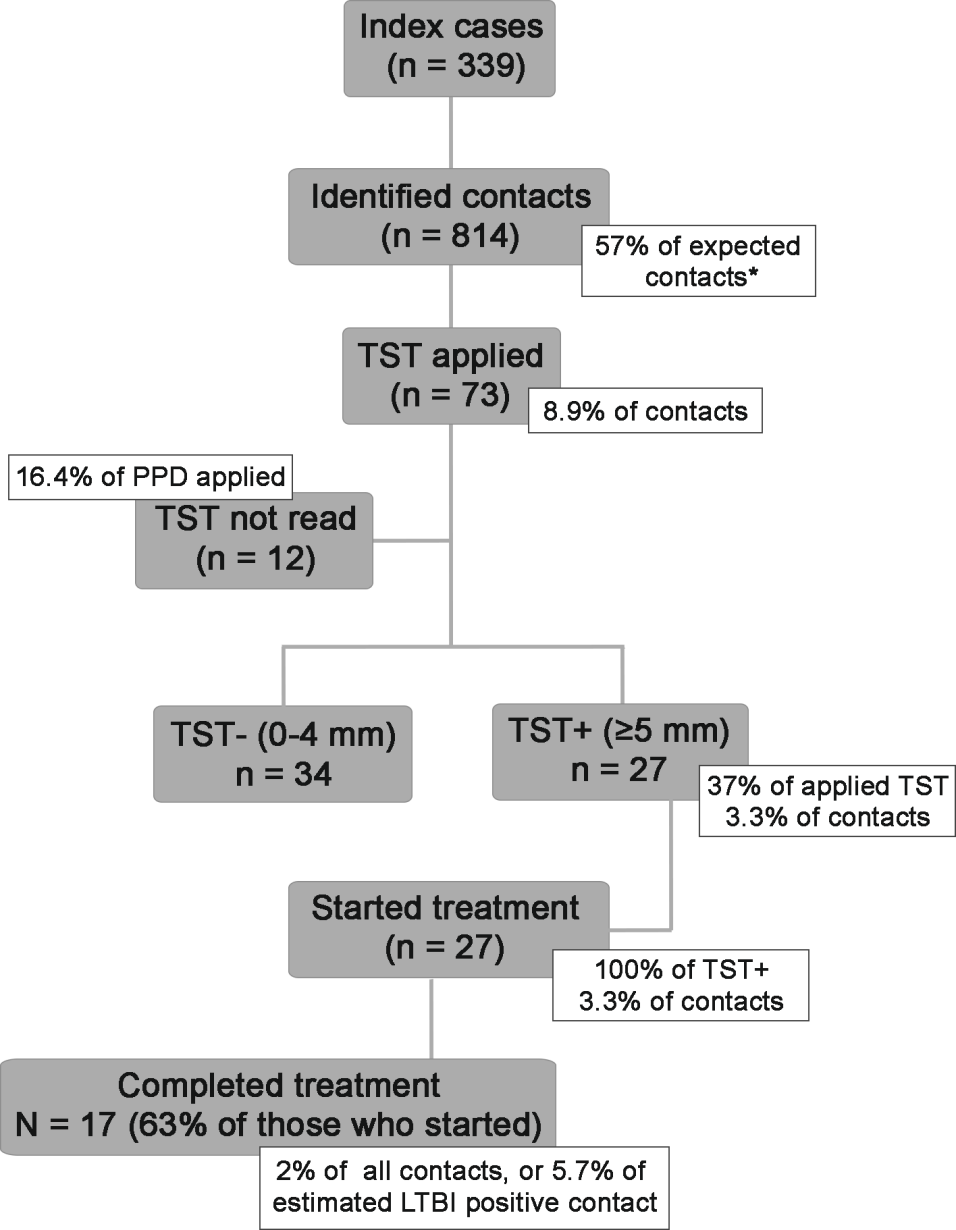
Cascade of latent tuberculosis infection care on the twelve participant health units in the Brazilian capitals, January-June 2014. Flowchart displaying the cascade of care of tuberculosis contacts in 12 clinics in Brazil, January-June 2014. * Expected contacts calculated multiplying the number of index cases by 4.2; TST = Tuberculin Skin Test.

Overall, 814 contacts from 339 registered index cases were identified (mean = 2.4 contacts/index case) in the records. Out of these, 73 (8.9%) underwent TST of whom 12 (16.4%) did not have TST read and 27 (36.9% of those read, or 3.3% of all contacts) were TST positive. All 27 started LTBI treatment, of whom 17 (62.9% of those who started treatment or 2.1% of all contacts and approximately 5.7% of all contacts estimated to have LTBI) completed treatment (Fig 1).

### Questionnaires

According to routinely collected data in the clinic, there were approximately 290 cases prospectively diagnosed in the 12 units during the interview study period, with approximately 410 close contacts, which constituted the eligible population for the interviews. They were approached if they were present on the day of data collection in their respective clinics. Among 138 index cases and 100 contacts across the three sites on the designated survey days, only two contacts refused to participate in the study. Eleven of 19 contacts were interviewed at home in Rio, two of 42 in Recife and none of 37 in Manaus. Their median age was 40 years [interquartile range (IQR) = 31 to 54.5] and 42 years [IQR = 30.5 to 53], respectively. The average duration of the interview with index cases was 10 minutes (IQR = 6 to 15) and with contacts, 11 minutes (IQR = 9 to 16).

Contacts´ knowledge and beliefs about TB are shown in Table 1. Knowledge about TB transmission and symptoms was satisfactory (67% and 87% respectively) and 28% knew how to prevent the disease. Half of them (52%) believed they could develop TB in the future, and 17% thought they were infected with *Mycobacterium tuberculosis*. Many (61%) believed they could spread TB to other people, except in Rio de Janeiro, where only 26% believed. When asked if they would take medicines to prevent TB if prescribed by the doctor, 81% answered positively. However, in Rio de Janeiro, only a minority (22%) said they would (Table 1).

**Table 1:** Knowledge and beliefs about transmission and prevention of tuberculosis among 98 contacts interviewed in Rio de Janeiro, Recife and Manaus, Brazil.

	Rio de Janeiro (n = 19)	Manaus (n = 37	Recife n = 42	Total
Answers	n = 19	n = 37	n = 42	n = 98
	n (%)	n (%)	n (%)	n (%)
How can a person get TB? *				
**Living in the same house with someone with TB^†^**	13(68)	7 (19)	5 (12)	25 (25)
**Contact with sick person through sneezing or coughing^†^**	5 (26)	25 (68)	30 (71)	60 (61)
**Sharing cutlery and dishes^‡^**	0 (0)	5 (14)	0 (0)	5 (5)
**God’s decision^‡^**	0 (0)	0 (0)	1 (2)	1 (1)
**I don’t know^‡^**	0 (0)	7 (19)	6 (14)	13 (13)
**Satisfactory answers^§^**	14 (74)	21 (57)	31 (74)	66 (67)
**Can TB be prevented?**				
**Yes**	13 (68)	20 (54)	36 (86)	69 (70)
**No**	6 (32)	17 (46)	6 (14)	29 (30)
How can TB be prevented? *				
**Taking prescribed medication^†^**	8 (42)	6 (16)	3 (7)	17 (17)
**Living in a big house with more rooms for the family^†^**	5 (26)	1 (3)	1 (2)	7 (7)
**Not getting close to the sick person^†^**	0 (0)	9 (24)	13 (31)	22 (22)
**Avoiding coughing and sneezing close to me^†^**	3 (16)	1 (3)	1 (2)	5 (5)
**No working/living in polluted enviroment^‡^**	0 (0)	1 (3)	9 (21)	10 (10)
**No sharing cutlery, glasses and dishes^‡^**	1 (5)	3 (8)	1 (2)	5 (5)
**Taking BCG vaccine^‡^**	0 (0)	1 (3)	1 (2)	2 (2)
**Performing sputum smear analysis^‡^**	1 (5)	1 (3)	4 (10)	6 (6)
**I don’t know^‡^**	5 (26)	7 (19)	4 (10)	16 (16)
**Satisfactory answers^§^**	3 (16)	8 (22)	8 (19)	19 (19)
What are the symptoms of pulmonary TB? *				
**Cough^†^**	15 (79)	28 (76)	32 (76)	75 (77)
**Tiredness^†^**	9 (47)	11 (30)	13 (31)	33 (34)
**Weight loss^†^**	11 (58)	10 (27)	27 (64)	48 (49)
**Fever^†^**	10 (53)	12 (32)	26 (62)	48 (49)
**Sputum with blood^†^**	8 (42)	7 (19)	7 (17)	21 (21)
**Night sweating^†^**	0 (0)	1 (3)	0 (0)	1 (1)
**Chest pain^‡^**	0 (0)	2 (5)	1 (2)	3 (3)
**Breathlessness ^‡^**	0 (0)	1 (3)	0 (0)	1 (1)
**Satisfactory answers^§^**	15 (79)	30 (81)	39 (93)	85 (87)
**Do you think you may have the TB bug? ^¶^**	n = 10	n = 35	n = 39	n = 84
**Yes**	0 (0)	4 (11)	10 (26)	14 (17)
**No**	8 (80)	29 (83)	27 (69)	64 (76)
**I don’t know**	2 (20)	2 (6)	2 (5)	6 (7)
**Are you afraid of having TB?**				
**Yes**	7 (37)	20 (54)	13 31)	40 (41)
**No**	12 (63)	17 (46)	29 (69)	58 (59)
**Do you think you may have TB in the future?**				
**Yes**	5 (26)	18 (49)	28 (67)	51 (52)
**No**	14 (74)	19 (51)	14 (33)	47 (48)
**Do you think you can spread TB to anyone?**				
**Yes**	5 (26)	27 (73)	28 (67)	60 (61)
**No**	14 (74)	10 (27)	14 (33)	38 (39)
If you have/had the infection and the doctor prescribes treatment, will/would you take it? **	n = 18	n = 36	n = 39	n = 93
**Yes**	4 (22)	33 (92)	38 (97)	75 (81)
**No**	14 (78)	3 (8)	1 (3)	18 (19)

TB = tuberculosis

* Multiple answers were allowed

^†^ One of the options necessary for a satisfactory answer

^‡^ None of these options must be selected so the response is classified as satisfactory

§ Knowledge was considered satisfactory if one of the options considered as necessary was mentioned by the respondent, since one of the options considered as excluding was not mentioned. The criteria to classify the answers as necessary or excluding response was done by a team of three experts

^¶^ Included only those who referred starting medicine fr TB/LTBI

** We excluded those who started treatment for LTBI.

Table 2 displays contacts´ perceptions of health services. Forty-three percent informed they had already performed or would perform TB tests. Among them, more were—or were scheduled to be—submitted to smear microscopy (22/42, 52%) and chest radiographs (20/42, 50%) than to TST (14/42, 33%). Only 21% reported that the health team alerted them that they could be sick or infected. Among the 56 contacts not investigated or scheduled for any investigation, the main reasons were believing they were not sick (42%) and tests not being proposed by the health team (36%). None of the respondents mentioned they could not afford the transportation costs and only one attributed lack of health-facility engagement to distance from their domicile.

**Table 2:** Perceptions and use of health services among 98 contacts interviewed in Rio de Janeiro, Recife and Manaus, Brazil.

	Rio de Janeiro	Manaus	Recife	Total
Answers	n = 19	n = 37	n = 42	n = 98
	n (%)	n (%)	n (%)	n (%)
**Performed or will perform any test**				
**Yes**	15 (79)	7 (19)	20 (48)	42 (43)
**No**	4 (21)	30 (81)	22 (52)	56 (57)
**Reasons to have gone to the health unit**				
**Make initial assessment for TB**	7 (37)	3 (8)	3 (7)	13 (13)
**I was not feeling well**	0 (0)	1 (3)	3 (7)	4 (4)
**I was afraid of having TB**	2 (11)	0 (0)	0 (0)	2 (2)
**The health team invited me**	14 (74)	2 (5)	1 (2)	17 (17)
**To support a family member or a friend**	2 (10)	27 (73)	32 (76)	61 (62)
**I had cough**	1 (5)	0(0)	1 (2)	2 (2)
Reasons for not having performed tests*^†^	n = 4	n = 30	n = 22	n = 56
**I did not believe I was sick**	1 (25)	11 (37)	13 (59)	25 (42)
**I was very busy**	0 (0)	1 (3)	1 (5)	2 (3)
**I do not like doctors / hospitals**	0 (0)	0 (0)	2 (9)	2 (3)
**There was no one to go with me**	0 (0)	0 (0)	1 (5)	1 (2)
**The health team has not requested exams for me**	2 (50)	13 (43)	6 (27)	21 (36)
**This unit is not equipped to take the exams**	1 (25)	0 (0)	0 (0)	1 (2)
**I have known recently about the diagnosis of IC**	0 (0)	2 (7)	2 (9)	4 (7)
**I could not afford transport costs**	0 (0)	0 (0)	0 (0)	0 (0)
**The health unit is too far from home**	0 (0)	1 (3)	0 (0)	1 (2)
**I don’t have free time at work/school**	0 (0)	0 (0)	0 (0)	0 (0)
**Procedures performed at the health unit ^‡^^†^**	n = 15	n = 7	n = 20	n = 42
**They asked about my symptoms**	9 (60)	3 (43)	1 (5)	9 (21)
**They performed a TST**	9 (60)	2 (29)	3 (15)	14 (33)
**They requested a chest radiograph**	12 (80)	2 (29)	6 (30)	20 (50)
**They requested a sputum analysis**	13 (87)	2 (29)	7 (35)	22 (52)
**They explained to me that I can have the infection**	8 (53)	6 (86)	5 (25)	19 (45)
**They explained to me that I might be sick**	5 (33)	1 (14)	3 (15)	9 (21)
**What kind of health facility would you look first if you were thinking you might be with TB?**				
**Primary care health center**	17 (90)	28 (76)	16 (38)	72 (73)
**Other sites**	2 (10)	9 (24)	26 (62)	26 (27)

TB = tuberculosis; IC = index case; TST = Tuberculin skin test

* Included only those who said they did not and they would not do exams

^†^ Multiple answers were accepted

^‡^ Included only those who said they had done or would do some exam.

About two thirds of contacts said they incurred out-of-pocket expenditures [at an average of 7.80 American dollars (USD); minimum wage in Brazil in 2015 = 236.56 USD] and 6% declared opportunity costs (i.e., absence from work/school had consequences), as displayed in Table 3.

**Table 3:** Out-of-pocket and indirect costs among 98 contacts interviewed in Rio de Janeiro, Recife and Manaus, Brazil.

	Rio de janeiro	Manaus	Recife	Total
Answers	n = 19	n = 37	n = 42	n = 98
	n (%)	n (%)	n (%)	n (%)
**Was it necessary that you lose your work day or school to go to the health unit?**				
**Yes**	8 (42)	3 (8)	9 (21)	20 (20)
**No**	5 (26)	17 (46)	17 (41)	39 (40)
**Not applicable**	6 (32)	17 (46)	16 (38)	39 (40)
There was some consequence because you have missed work or school to come to the health facility? *	n = 8	n = 3	n = 9	n = 20
**Yes**	4 (50)	0 (0)	2 (22)	6 (30)
**No**	4 (50)	3 (100)	7 (78)	15 (70)
**Time and costs involved to get to the unit**				
**Time spent on travel (round trip, in minutes). Average (± SD)**	43 (±38)	89 (±89)	51 (±35)	64 (±64)
**Waiting time at the facility (waiting and consultation in minutes). ^†^ Average (± SD)**	68 (±53)	7 (±23)	11 (±33)	14 (±33)
**Spending money on food and transport: Average (± SD)**	R$ 9.50 (±14) USD 2.87 (±4)	R$ 19.58 (±24) USD 5.91 (±7)	R$ 38.70 (±87) USD 11.69 (±26)	R$ 25.82 (±60) USD 7.80 (±18)
**Reported expenses for food and/or transportation (%)**	9 (47)	32 (87)	28 (67)	69 (70)

SD = standard deviation; R$ = Brazilian Reais; USD = American dollar (conversion to USD considering the average price for purchase of USD in 2015)

* Not included those who declared themselves as unemployed and retirees

^†^ Included only those who said they had done or would do some exam.

There were no significant differences in knowledge or attitudes related to LTBI investigation and treatment according to sex, schooling and city, apart from the specificities in Rio described above (Table 4).

**Table 4:** Comparative proportions of satisfactory answers according to the characteristics of 98 contacts interviewed in Recife, Rio de Janeiro and Manaus, Brazil.

Questions	Satisfactory answers n/total (%)	p-value
**Sex**	66/98 (67)	0.413*
Male	15/66 (23)	
Female	51/66 (77)	
**Year of education**	66/98 (67)	0.971*
> 4 years	62/66 (94)	
< 4 years	4/66 (6)	
**City**	66/98 (67)	0.220^**†**^
Manaus	21/37 (57)	
Recife	31/42 (74)	
Rio de Janeiro	14/19 (74)	
**Sex**	69/98 (70)	0.109*
Male	17/69 (25)	
Female	52/69 (75)	
**Year of education**	69/98 (70)	0.836*
> 4 years	65/69 (94)	
< 4 years	4/69 (6)	
**City**	69/98 (70)	0.009^**†**^
Manaus	20/37 (54)	
Recife	36/42 (86)	
Rio de Janeiro	13/19 (68)	
**How TB can be prevented?**		
**Sex**	19/98 (19)	0.938*
Male	4/19 (21)	
Female	15/19 (79)	
**Year of education**	19/98 (19)	0.215*
> 4 years	19/19 (100)	
< 4 years	0/19 (0)	
**City**	19/98 (19)	0.870^**†**^
Manaus	8/37 (22)	
Recife	8/42 (19)	
Rio de Janeiro	3/19 (16)	
**What are the symptoms of pulmonary TB?**		
**Sex**	85/98 (87)	0.798*
Male	17/85 (20)	
Female	68/85 (80)	
**Year of education**	85/98 (87)	0.323*
> 4 years	79/85 (93)	
< 4 years	6/85 (7)	
**City**	85/98 (87)	0.286^**†**^
Manaus	30/37 (81)	
Recife	39/42 (93)	
Rio de Janeiro	16/19 (84)	

* p-value by Chi-square test

† p-value by Chi Square for linear trend (Extended Mantel-Haenszel).

Table 5 displays knowledge and beliefs about TB reported by index cases. Seventy-three percent declared they feared dying from TB, 67% believed they could transmit TB, but only 23% understood how they got the disease. The main misconceptions regarding transmission included having inadequate nutrition, drinking alcohol, smoking, exposure to pollution and overwork. As for their attitudes, 94% said they had shared or would share with their families the diagnosis of TB and 88% would like their family members to be investigated for TB and LTBI. Fear of social rejection was mentioned by 10% of the cases. Most were satisfied with the clinics. There were no major differences in the number of satisfactory answers according to the variables analyzed in the present study (sex, city, schooling and family income, Table 6).

**Table 5:** Knowledge, attitudes and beliefs about tuberculosis and perceptions of health services among the 138 index cases in Rio de Janeiro, Manaus and Recife, Brazil.

	Rio de Janeiro	Manaus	Recife	Total
Answers	(n = 28)	(n = 61)	(n = 49)	(n = 138)
	n (%)	n (%)	n (%)	n (%)
**Do you think you can transmit TB to another person?**				
**Yes**	20 (72)	42 (69)	31 (63)	93 (67)
**No**	6 (21)	11 (18)	18 (37)	33 (24)
**I don’t know**	2 (7)	8 (13)	2 (4)	12 (9)
**Do you think you can die because of TB?**				
**Yes**	19 (68)	48 (79)	33 (67)	100 (73)
**No**	8 (29)	10 (16)	14 (29)	32 (23)
**I don’t know**	1 (3)	3 (5)	2 (4)	6 (4)
How do you think got TB? *				
**Satisfying answers^†^**	11 (39)	14 (23)	7 (14)	32 (23)
**You will say or told your family that you got TB?**				
**Yes**	25 (89%)	57 (93%)	48 (98%)	130 (94%)
**No**	3 (11%)	4 (7%)	1 (2%)	8 (6%)
**Would you like people who live with you being examined to check if any of them got TB?**				
**Yes**	21 (75%)	57 (93%)	48 (98%)	126 (91%)
**No**	7 (25%)	4 (7%)	1 (2%)	12 (9%)
What is your biggest concern in the case of other people in your family get sick of TB in the future? *				
**Concern about their health**	19 (68)	43 (71)	35 (71)	97 (70)
**I fear they could die**	0 (0)	9 (15)	8 (16)	17 (12)
**I'm not worried**	2 (7)	2 (3)	4 (8)	8 (6)
**Social rejection**	3 (11)	7 (11)	4 (8)	14 (10)
**I'm afraid they cannot take care of me**	0 (0)	3 (5)	4 (8)	7 (5)
**They may lose their jobs**	2(7)	3 (5)	2 (4)	7 (5)
**Others**	3 (11)	1 (2)	3 (6)	7 (5)
**Are you satisfied with the information you have about your illness?**				
**Yes**	25 (89)	52 (85)	46 (94)	123 (89)
**No**	3 (10)	9 (15)	3 (6)	15 (11)
**Are you satisfied with the care in the unit?**				
**Yes**	26 (93)	58 (95)	45 (92)	129 (94)
**No**	2 (7)	3 (5)	4 (8)	9 (6)

TB = tuberculosis; HIV = Human immunodeficiency virus

* Multiple answers were allowed

^†^ The answer was considered satisfactory if one of the options considered as necessary was mentioned by the respondent, since one of the options considered as excluding was not mentioned. The criteria to classify the answers as necessary or excluding response was done by a team of three experts.

**Table 6:** Comparative proportions of satisfactory answers according to the characteristics of the 138 index cases interviewed in Recife, Rio de Janeiro and Manaus, Brazil.

Questions	Satisfactory answers n/total (%)	p-value
**How do you think you got TB?**		
**Sex**	32/138 (23)	0.044*
Male	11/32 (34)	
Female	21/32 (66)	
**Years of education**	32/138 (23)	0.294*
> 4 years	29/32 (90)	
< 4 years	3/32 (10)	
**City**	32/138 (23)	0.368**†**
Manaus	14/32 (44)	
Recife	7/32 (22)	
Rio de Janeiro	11/32 (34)	
**Will you tell or have told your family about your disease?**		
**Sex**	130/138 (94)	0.145*
Male	63/130 (49)	
Female	67/130 (51)	
**Years of education**	130/138 (94)	0.427*
> 4 years	111/130 (85)	
< 4 years	19/130 (15)	
**City**	130/138 (94)	0.505**†**
Manaus	57/130 (44)	
Recife	48/130 (37)	
Rio de Janeiro	25/130 (19)	
**Would you like people who live with you to be examined to check if any of them got TB?**		
**Sex**	126/138 (91)	0.009*
Male (n = 69)	58/126 (46)	
Female (n = 69)	68/126 (54)	
**Years of education**	126/138 (91)	0.323*
> 4 years	108/126 (86)	
< 4 years	18/126 (14)	
**City**	126/138 (91)	0.036**†**
Manaus	57/126 (45)	
Recife	48/126 (38)	
Rio de Janeiro	21/126 (17)	
**What is your biggest concern in the case of other people in your family getting sick with TB in the future?**		
**Sex**	102/138 (74)	0.438*
Male	49/102 (48)	
Female	53/102 (52)	
**Years of education**	102/138 (74)	0.181*
> 4 years	84/102 (82)	
< 4 years	18/102 (18)	
**City**	102/138 (74)	0.971**†**
Manaus	46/102 (45)	
Recife	38/102 (37)	
Rio de Janeiro	18/102 (18)	

* p-value by Chi-square test

† p-value by Chi Square for linear trend (Extended Mantel-Haenszel)

## Discussion

In the present study, designed to understand where (cascade) and why (interviews) losses in the cascade of LTBI care occur, we explored the users’ perspective. We observed very low rates of LTBI investigation among contacts as observed in other countries.[8,32–34] Losses occurred mainly in the first steps–identification and investigation–and this was confirmed by the questionnaires. Indeed, much more contacts (4.2 contacts/index case) were expected.[35] More importantly, the great majority of identified contacts were never investigated for LTBI. Losses in the first steps were recognized in a systematic review both in high- and in low- and medium-income countries,[8] but the magnitude of these losses in the current study were much higher. Many TB cases could be avoided in Brazil if all contacts were investigated.[36]

Interestingly, however, the majority of contacts would like to have been investigated and would take preventive therapy if prescribed, which represents, in this case, a lost opportunity. Likewise, their index case would like them to be investigated and treated for TB or LTBI. In contrast, healthcare workers’ in Brazil reported perceptions that contacts wouldn’t or do not engage TB services because they do not understand the importance of TB or because index cases fear stigma and do not share their diagnosis with their contacts.[21] Their perceptions are not supported by the present study.

Contacts who did undergo evaluation were mostly investigated for active TB, seldom for LTBI. Indeed, sputum smear and chest radiographs were performed for over half the contacts that attended clinics. Our cascade refers to the period when PPD was fully available in the international market. Thus, barriers for contact investigation in Brazil go beyond the availability of PPD.

The reasons for low rates of contact investigation for LTBI do not appear to rest with the contacts themselves or their index cases in Brazil. They have a reasonable knowledge of their risk, they do fear TB–unlike reports from other populations-[15,37] and most understand the disease can be prevented. Furthermore, index cases fear for their family, they would like their contacts to be investigated and treated, and recognize the importance of these procedures. Fear of social rejection or stigmatization was not an issue, unlike reported in other studies.[15,17] Moreover, they are satisfied with the services they attend and costs do not seem to be a barrier. Thus, overall, both index cases and contacts had a good attitude towards LTBI investigation and treatment.

Why then are so few contacts investigated in clinics in Brazil? According to their view, one of the main reasons was they were not invited by the health team, a finding reported in previous studies.[38] We are currently investigating knowledge, attitudes and practices of healthcare workers in the same clinics in order to have a broader picture, with different perspectives.

Contacts in Rio had a different perception and were less knowledgeable compared to those in the two other cities. At least in part, this is likely to reflect that the majority of contacts in Rio were interviewed at home. Contacts that do not go to the clinics may well be different from those who attend visits either with their index case or after responding to the request of a healthcare worker. Our study has other limitations. In-depth understanding of reasons for an observed phenomenon usually needs qualitative methods. We did not explore factors linked to knowledge, attitudes and beliefs based on theoretical models. This was outside the scope of the present research. In addition, in studies based on interviews, respondents may not feel confident about responding to questions they think could be disapproved by the interviewer or the clinic staff, a potential source of bias. However, interviewers in this study were not part of the clinic staff. Finally, the sample is small and does not include children, although the questionnaire contained questions regarding close contact children and families with and without children were included in the sample.

In summary, there is a very low rate of investigation for LTBI of TB contacts in three high burden cities in Brazil. Contacts’ knowledge, attitudes and service perceptions do not seem to be the main reason for this finding. On the contrary, their will to be treated should be better explored by the health team and TB programs. Contact and index case education would not be enough to overcome the barriers. We suggest that interventions should be tailored to local values and cultures, which may vary even in the same country. The findings of the present study will be presented to TB managers and clinic staff in each unit in order to collectively find reasonable, feasible and sustainable solutions.

## Supporting information

S1 Table Database of the contacts interviewed during the survey. (XLSX)

S2 Table Database of the index cases interviewed during the survey. (XLSX)
